# Comparing dose response of cancer incidence in Sweden after the Chernobyl Nuclear Power Plant accident with Life Span Study of atomic bomb survivors

**DOI:** 10.1093/rpd/ncaf097

**Published:** 2025-08-21

**Authors:** Martin Tondel, Tobias Nordquist, Mats Isaksson, Christopher Rääf, Robert Wålinder

**Affiliations:** Occupational and Environmental Medicine, Department of Medical Sciences, Uppsala University, Uppsala University Hospital, Entrance 40, SE-751 85 Uppsala, Sweden; Department of Occupational and Environmental Medicine, Uppsala University Hospital, Dag Hammarskjölds väg 60, SE-751 85 Uppsala, Sweden; Department of Occupational and Environmental Medicine, Uppsala University Hospital, Dag Hammarskjölds väg 60, SE-751 85 Uppsala, Sweden; Department of Medical Radiation Sciences, Institute of Clinical Sciences, Sahlgrenska Academy, University of Gothenburg, Gula Stråket 2B, SE-413 45, Gothenburg, Sweden; Medical Radiation Physics, Department of Translational Medicine, Lund University, Inga Marie Nilssons gata 47, SE-205 02 Malmö, Sweden; Occupational and Environmental Medicine, Department of Medical Sciences, Uppsala University, Uppsala University Hospital, Entrance 40, SE-751 85 Uppsala, Sweden; Department of Occupational and Environmental Medicine, Uppsala University Hospital, Dag Hammarskjölds väg 60, SE-751 85 Uppsala, Sweden

## Abstract

Follow-up of the atomic bomb survivors in Japan in the Life Span Study (LSS) has been fundamental for the understanding of the dose–response curve. We compare our risk estimates from a newly published epidemiological study on cancer in Sweden after the Chernobyl Nuclear Power Plant accident with the LSS data. Hazard ratios (HRs) with 95% confidence intervals (95% CIs) were calculated using conditional logistic regression adjusted for rural/nonrural habitat, education level, and pre-Chernobyl cancer incidence from 1980 to 1985, respectively. Adjusted HRs by sex were calculated in deciles for all cancer sites combined for 1986 to 2020. These risk estimates were translated to excess relative risk (ERR) to allow comparison with LSS incidence data. ERRs per decile were compatible with ERR in the low-dose range <100 mGy for both sexes. The CIs in each decile need to be taken into account when interpreting the dose–response curve. Risk estimates in dose categories add important information at very low doses on the dose–response curve when compared to LSS data.

## Introduction

Our knowledge on the cancer dose–response curve of ionizing radiation is highly influenced by the follow-up of the atomic bomb survivors in Japan, the Life Span Study (LSS). Several evaluations of the epidemiological studies have supported a linear no-threshold (LNT) model for radiation protection purposes, such as the International Commission on Ionizing Radiation (ICRP), National Research Council (BEIR VII), United Nations Scientific Committee on the Effects of Atomic Radiation (UNSCEAR), and US National Council on Radiation Protection and Measurements (NCRP) [1–4]. The ICRP has raised the importance of studying the dose–response curve at low doses in evaluating the LNT [5]. Wakeford *et al.* (2023) recently also pointed out the importance to continually review the LNT model in the context of new evidence [6]. Earlier attempts to study the dose response at low radiation exposure levels in the LSS have shown statistically significant risk on the ‘incidence’ of solid cancer <100 mSv [7] and on ‘mortality’ of solid cancer compatible with LNT at a low dose, i.e. <200–300 mGy [8]. Later analysis has focused on the dose response of incident solid cancer with statistically significant increase in dose categories for females from 125 to 175 mGy and nonsignificant increase from 20 to 125 mGy [9]. For males this relationship was a statistically nonsignificant increase in the dose categories < 300 mGy [9]. In the latest follow-up of the LSS, solid cancer incidence was studied at doses < 100 mGy, with an estimated excess relative risk (ERR) of 0.32/Gy for males and 0.40 for females [10]. However, the exposure to the atomic bomb survivors was a single and comparatively high acute exposure not necessarily applicable in a situation of protracted exposure at very low doses. After the Chernobyl Nuclear Power Plant (NPP) accident in Ukraine in 1986, we performed several epidemiological studies of cancer in Sweden [11–15]. In our latest follow-up of incident cancer up to 2020, we could for the first time study the dose–response curve in detail using estimated whole body doses in milligray (mGy) [16]. The absorbed external and internal doses are estimated from the Chernobyl fallout in Sweden and are additional to the natural background radiation (terrestrial gamma radiation, indoor radon exposure) and medical X-ray examinations. We hereby aim to put our reported risk estimates in the low dose range 0–10 mGy in relation to the LSS risk estimates at low doses of 0–100 mGy.

## Materials and methods

**Figure 1:**
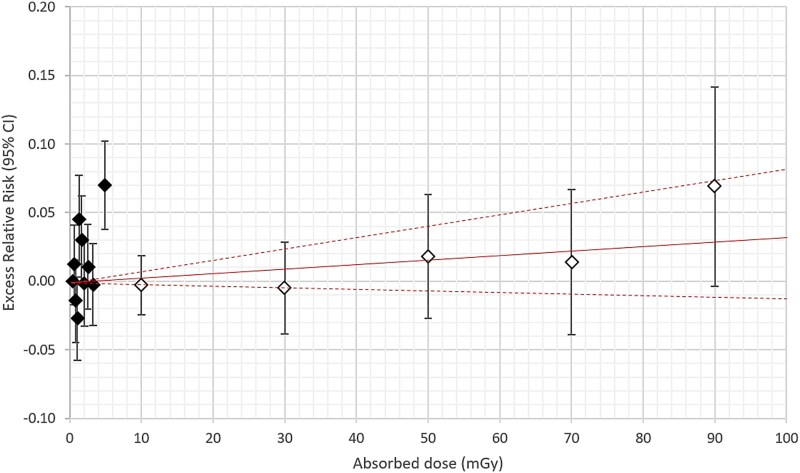
Male. Excess relative risk (ERR) with 95% confidence interval (95% CI) in vertical bars for all solid cancer incidence from appendix table E in Grant *et al.* (2017) (unfilled diamonds) compared with all cancer incidence from Tondel *et al.* (2023) (filled diamonds) [9, 16]. All ERR estimates are put at the average in each absorbed dose category. The solid line is ERR per milligray with 95% CI (dashed line) according to Brenner *et al.* [10]. The dose range is 0–100 mGy for both comparisons.

**Figure 2:**
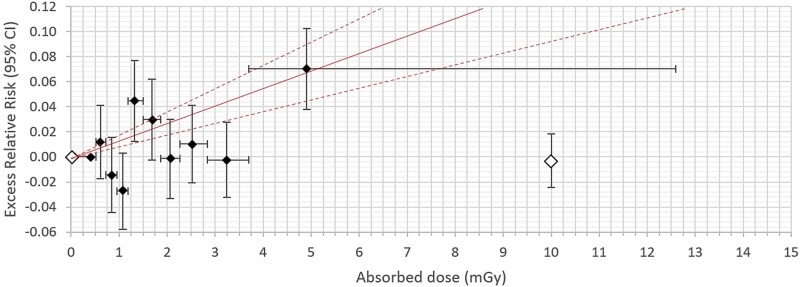
Male. Excess relative risk (ERR) with 95% confidence interval (95% CI) in vertical bars for all solid cancer incidence from appendix table E in Grant *et al.* (2017) (unfilled diamonds) compared with all cancer incidence from Tondel *et al.* (2023) (filled diamonds) [9, 16]. Horizontal bars show the dose range in each decile with ERR at the average of the absorbed dose in each decile. The solid line is ERR per milligray with 95% CI (dashed line) according to Tondel *et al.* [16]. The dose range is 0–15 mGy for both comparisons.

**Figure 3:**
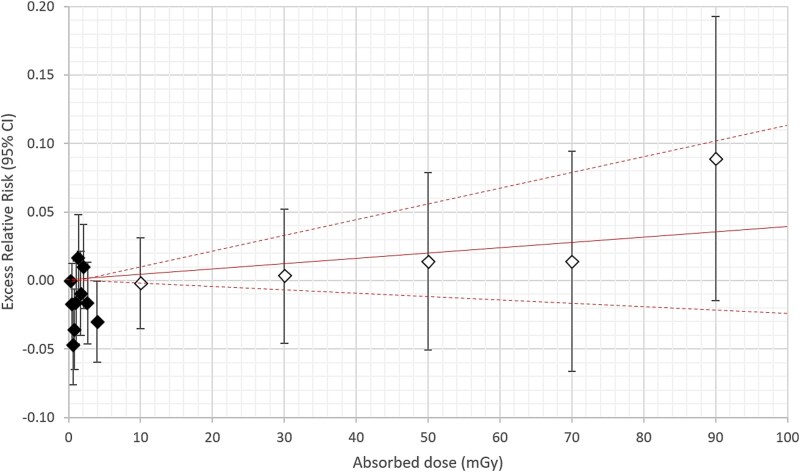
Female. Excess relative risk (ERR) with 95% confidence interval (95% CI) in vertical bars for all solid cancer incidence from appendix table E in Grant *et al.* (2017) (unfilled diamonds) compared with all cancer incidence from Tondel *et al.* (2023) (filled diamonds) [9, 16]. All ERR estimates are put at the average in each absorbed dose category. The solid line is ERR per milligray with 95% CI (dashed line) according to Brenner *et al.* [10]. The dose range is 0–100 mGy for both comparisons.

**Figure 4:**
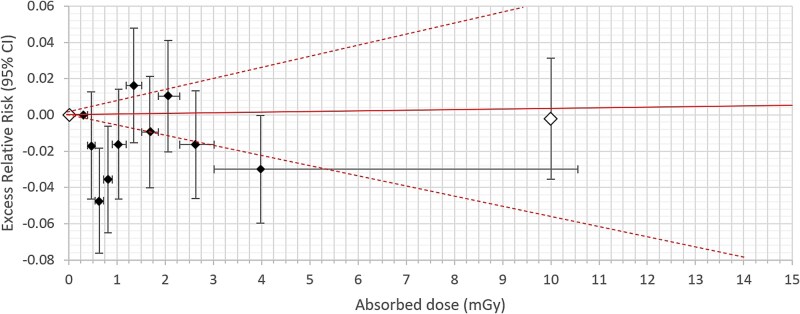
Female. Excess relative risk (ERR) with 95% confidence interval (95% CI) in vertical bars for all solid cancer incidence from appendix table E in Grant *et al.* (2017) (unfilled diamonds) compared with all cancer incidence from Tondel *et al.* (2023) (filled diamonds) [9, 16]. Horizontal bars show the dose range in each decile with ERR at the average of the absorbed dose in each decile. The solid line is ERR per milligray with 95% CI (dashed line) according to Tondel *et al.* [16]. The dose range is 0–15 mGy for both comparisons.

From our recent publication we have chosen to use the results from the *post hoc* analysis on seven counties as we consider these risk estimates less sensitive to remaining uncontrolled confounding, compared to the full analysis of nine counties [16]. A nested case control methodology was chosen due to the protracted dose during the follow-up period. In our study, each incident cancer case was matched to four living controls using sex, year of diagnosis, and year of birth ± 2 years as matching variables. Hence, the absorbed dose to the controls represent the exposure to a person not retrieving cancer at the same time the case got diagnosed. By randomly selecting controls from the source population, in our case the closed cohort, the controls (representing the dose distribution in the population) can by statistical reason have a higher or lower absorbed dose than the cancer case. The absorbed dose to a control then represents the dose a control would have had if it had become a case at the same time the matched case got its cancer diagnosis. If cases on average have a slightly higher absorbed dose than controls, it indicates that radiation is a risk factor for cancer. Here we only present adjusted hazard ratios (adj HRs) for incidence of cancer at all sites taken together and statistically adjusted for potential confounding from urban lifestyle in 1986, the socioeconomic status defined as the highest educational level attained during follow-up to 2020 and pre-Chernobyl cancer incidence by cancer site from 1980 to 1985, respectively. The rationale for and definition of the potential confounding factors are described in Tondel *et al*. [16]. These HRs are calculated in deciles using the first decile as the reference category. Our deciles are presented in the graphs at the average of the absorbed dose in each decile and with horizontal bars showing the dose range in each decile (Figs 1–4). As we used a Cox proportional hazard survival model with fixed time to calculate the HR, it equals the RR [17]. In our study the HRs with 95% confidence intervals (95% CIs) were calculated in a conditional logistic regression with matched pairs (four controls matched to each cancer case) applying strata for date of diagnosis, age, and sex, in the PHREG procedure in SAS, version 9.4 [16, 17]. The ERR in each decile can then be obtained by subtracting 1 from our adj HR and from the upper and lower CI, hence resulting in an ERR with 95% CI. For all cancer sites considered together, adj HRs were also calculated using a linear model with total absorbed dose per milligray as a continuous variable, expressed as adj HR per milligray. Similarly, the ERR per milligray was obtained by subtracting 1 from our adj HR and the corresponding CI.

For comparison, a sex-specific ERR with 95% CI for all solid cancer incidence was retrieved from table E in the appendix in Grant *et al.* and the ERRs (95% CI) were, like our own data in the graphs, put at the average absorbed dose in each dose category. These average values for each exposure category were also retrieved from table E [9]. As Grant *et al*. did not give an exact lower CI, we used the normal approximation to calculate the upper and lower CI (ERR +/− 1.96 × SE) [9]. In addition, ERRs per milligray were retrieved from table 4 in Brenner *et al.* for solid cancer incidence at doses <100 mGy by dividing the ERR per gray and the corresponding CI by 1000 [10].

## Results

ERRs with 95% CIs are presented for males and females separately, in Figs 1–4, respectively. The average absorbed dose in the lowest dose category 5–20 mGy was 10 mGy in the LSS data for both sexes [9]. Hence, the dose range in LSS at 10 mGy partly overlapped our highest decile dose range for males of 3.70–12.6 mGy and for females of 3.02–10.6 mGy, respectively (Figs 2 and 4). Note that the ERR for males in the highest decile is elevated, but with overlapping confidence intervals with other deciles from our study. The ERR per milligray (95% CI) estimated for males is 0.01402 (0.00939 to 0.01866) and for females is 0.00032 (−0.00568 to 0.00637) [16]. The corresponding ERR per milligray < 100 mGy in the LSS is 0.00032 (−0.00012 to 0.00085) for males and 0.00040 (−0.00025 to 0.00115) for females [10].

## Discussion

Our results in cancer incidence expressed as ERR by deciles in Sweden after the Chernobyl NPP accident are in the same range as the LSS data. However, applying a linear function as ERR per milligray from our Chernobyl study compared with the LSS shows a more steep dose–response relationship in males, but in the same range in females below 15 mSv, compared to the latest update from the LSS [10]. As our dose range is so narrow, and volatile for small changes in individual dose estimations, we have chosen to express ERR per milligray, instead of ERR per gray, to avoid magnifying the uncertainties inherited at low doses. Therefore, we also show our linear estimate only in Figs 2 and 4 as it would misrepresent our data to expand outside the dose above 15 mSv as we cannot know for certain if the linearity extend beyond these extremely low doses. Moreover, there is a scientific debate on the shape of the dose–response curve at low doses with different options presented, including the difficulties in establishing such a dose–response curve because of statistical limitations, misclassification of exposure, and potential confounding factors obfuscating the curvature [2, 9, 18, 19]. We believe our results could contribute to this discussion. However, using a linear function has a benefit as it makes it possible to compare the risk estimates between epidemiological studies, but we have here shown that caution is needed in the interpretation of results if ERR per milligray is the only way of expressing the risk. We could show that analysis by deciles adds important information when presenting the dose–response curve, ignored if results only are expressed as ERR per milligray. However, the CIs need to be taken into account when interpreting each decile.

We compare cancer of all sites of cancer after the Chernobyl NPP accident, with solid cancer in the LSS, but as solid cancer in our material constitutes >91% of all cancer it is of negligible importance in explaining the differences seen in this comparison. A nondifferential misclassification of exposure is random in nature i.e. tends to make the relation between exposure and effect weaker instead of creating spurious associations [20]. A more important limitation of our study is the risk of differential misclassification of absorbed doses, at an individual level, with an estimated uncertainty of up to 50% (at 1-sigma level) [21–23]. Previous assessments of uncertainty in our studies have given that the main sources of uncertainty are the transfer function, relating body burden of radiocaesium to the ground deposition, and the ecological half-life modelled by the *r*(*t*) function, relating the decrease in external exposure to weathering processes [21–23]. Due to limitations inherent in all low-dose-radiation epidemiological studies, it will probably not be possible to finally judge whether the dose–response curve is supralinear, linear, linear quadratic, or consistent with a threshold at extremely low doses [8, 18, 19, 24]. Comparing studies is a helpful tool to gain better knowledge on the dose–response curve. We cannot give a final judgement whether our results are influenced by uncontrolled confounding such as medical X-ray examinations associated with the Chernobyl fallout or socioeconomic factors not fully taken into account in our epidemiological modelling. A strength of our study is that it is unlikely that age asserts any confounding since each cancer case was matched to four controls for year of birth. Another strength is that we have applied an established statistical method using a standard procedure in the SAS program [17]. Expressing the cancer risk as ERR per milligray, justified by the LNT model, has the advantage of using the full range of exposure data, compared to apply a trend line only using the values of the deciles, but still puts limitations on interpretation. We encourage more researchers to study cancer incidence at very low doses in attempts to challenge the limitations pointed out in this paper. Such results can be used in meta-analysis to overcome lack of statistical power at low risks. Moreover, protracted exposure, including both external and internal absorbed doses, e.g. after the Chernobyl NPP accident, might yield different risks in comparison with a prompt exposure of neutron and gamma radiation studied in atomic bomb survivors in the LSS. The dose–response curve at very low doses is very important in radiation protection purposes because a small increment could potentially give rise to a substantial number of cancer cases as the majority of the population will be exposed to low doses in case of a future radionuclear incident. On the other hand, if such increment cannot be determined it could also influence policy in radiological protection.

## Data Availability

For this study we have only used published results from Grant *et al.*, Tondel *et al*., and Brenner *et al.*, respectively [9, 10, 16]. The primary dataset for our study is not publicly available due to privacy reasons and a nondisclosure statement to the National Board of Health and Welfare providing us with the diagnosis of cancer [16].
